# Systemic immune-inflammation index predicts prognosis of sequential therapy with sorafenib and regorafenib in hepatocellular carcinoma

**DOI:** 10.1186/s12885-021-08124-9

**Published:** 2021-05-18

**Authors:** Young Mi Hong, K. T. Yoon, Mong Cho

**Affiliations:** Department of Internal Medicine, Liver Center, Pusan National University School of Medicine, Pusan National University Yangsan Hospital, 20 Geumo-ro, Gyeongnam 50612 Yangsan, South Korea

**Keywords:** Regorafenib, Hepatocarcinoma carcinoma, Inflammation, Prognosis, Systemic immune-inflammtaion index, Alpha-fetoprotein

## Abstract

**Background:**

Regorafenib has shown promising results as a second-line therapy for patients with hepatocellular carcinoma (HCC) who progressed on sorafenib. Although there have been several data regarding the efficacy of sequential therapy with sorafenib and that of regorafenib in real-life, specific inflammation markers for predicting the prognosis have not been studied. This study aimed to investigate prognostic value of systemic inflammatory markers in patients with HCC who received sorafenib-regorafenib sequential therapy.

**Methods:**

We retrospectively analyzed medical data of patients who received regorafenib for the treatment of HCC after sorafenib failure. Progression free survival (PFS) and overall survival (OS) were assessed using the Kaplan–Meier survival curves. Univariate and multivariate analyses were performed to analyze the factors associated with survival.

**Results:**

A total of 58 patients who received at least one dose of regroafenib and fulfilled the eligibility criteria, good performance status (Eastern Cooperative Oncology Group [ECOG] 0–1) and preserved liver function (Child-Pugh-A), were included in the analysis. The median PFS was 3 months (95% confidence interval [CI] = 0.981–5.019) and the median OS was 8 months (95% CI = 5.761–10.239). Elevated systemic immune-inflammation index (SII ≥340) was independently associated with poor OS. In multivariate analysis, the SII (hazard ratio [HR] = 2.211, 95% CI = 1.089–4.489, *P* = 0.028) and alpha-fetoprotein (AFP) (HR = 2.750, 95% CI = 1.259–6.010, *P* = 0.011) were independent predictors of OS.

**Conclusion:**

Elevated SII is associated with poor OS in patients with HCC who received sequential therapy with sorafenib and regorafenib. In addition, when selecting a treatment strategy, the SII can be used in combination with the AFP level as a promising prognostic tool for HCC.

## Background

Hepatocellular carcinoma (HCC), one of the most common cancers, is the third leading cause of cancer-related mortality worldwide [1]. Despite the advancements in diagnosis and surveillance programs for HCC, the majority of patients are diagnosed in an advanced stage, thus being unsuitable to potentially curative treatments such as surgery, liver transplantation, or ablative therapy. Systemic treatment is recommended for patients with advanced HCC at diagnosis or with unresectable HCC who cannot benefit from locoregional therapy. Sorafenib, a multikinase inhibitor, is the first approved systemic chemotherapy for advanced HCC [2]. Since the approval of sorafenib in 2008, no second-line agents following sorafenib treatment were available for a fairly long time. Recently, regorafenib [3], cabozantinib [4], nivolumab [5], pembrolizumab [6], and ramucirumab [7, 8] have been approved as a second-line systemic chemotherapies for advanced HCC.

Regorafenib is an oral multikinase inhibitor that targets vascular endothelial growth factor receptors (VEGFRs) 1–3, KIT, RET, RAF-1, BRAF, platelet-derived growth factor receptor (PDGFR), fibroblast growth factor receptor (FGFR), and colony-stimulating factor 1 receptor (CSF1R) [9–11]. It has been adopted as an initial second-line agent and sorafenib-regorafenib sequential treatment in patients with advanced HCC. Although several studies have reported outcomes of sorafenib-regorafenib sequential treatment, few studies have been evaluated the prediction of survival.

Inflammation plays an essential role in tumor development and tumor metastasis, and immune surveillance plays a crucial role in cancer elimination [12, 13]. Recently, many studies have reported that combined scores using peripheral inflammatory cells (neutrophils, lymphocytes, and platelets) are associated with survival in various tumors [14–17]. To the best of our knowledge, however, no studies regarding systemic inflammatory markers in predicting HCC patients received sequential therapy with sorafenib and regorafenib. Therefore, we aimed to determine the prognostic value of systemic inflammatory markers in patients with advanced HCC.

## Methods

### Patient eligibility and regorafenib treatment

We retrospectively reviewed the data of patients who received regorafenib between July 2017 and April 2020. The diagnosis of HCC was based on American Association for the Study of Liver Diseases criteria. Patients who failed sorafenib treatment, which was confirmed based on radiological progression during sorafenib therapy, were enrolled. Inclusion criteria were in accordance with phase 3 trial of regorafenib (RESORCE trial) and were as follows: tolerability to sorafenib (≥400 mg daily for at least 20 of the 28 days) and preserved liver function (Child Pugh A). Regorafenib was prescribed in a 4-week cycles, with a starting dose of 160 mg once per day for 3 weeks and 1 week of no treatment. Dose reductions and interruption of regorafenib were performed based on occurrence of adverse events [3]. Regorafenib treatment was continued until patients experienced intolerable adverse events or until confirmation of radiologic progressive disease (PD). This retrospective study was reviewed and approved by the Ethical Committee of our center.

### Clinical variables

Clinical data such as baseline demographics, Eastern Cooperative Oncology Group performance status, complete blood count, alpha-fetoprotein (AFP), protein induced by vitamin K absence or antagonist II (*PIVKA*-*II*), Child-Pugh score, tumor stage, adverse events after regorafenib treatment, and radiologic assessment for tumor response were extracted. Adverse events were evaluated using National Cancer Institute Common Terminology Criteria for Adverse Events (NCI-CTCAE), version 4.03. Tumor response was evaluated based on radiological assessments such as computed tomography (CT) or magnetic resonance imaging (MRI) using Response Evaluation Criteria In Solid Tumors (RECIST) version 1.1. All laboratory parameters were assessed at the start of regorafenib treatment. Further, inflammatory markers, namely neutrophil to lymphocyte ratio (NLR), platelet to lymphocyte ratio (PLR), systemic immune-inflammation index (SII), were evaluated. The NLR was defined as the neutrophil count divided by the lymphocyte count. The PLR was defined as the platelet counts divided by the lymphocyte counts. The SII was calculated as platelet count × neutrophil count/lymphocyte count [18]. The NLR, PLR and SII scores were stratified into two groups based on each median value.

### Statistical analysis

Categorical variables were analyzed using Pearson’s chi-square test. Overall survival (OS) was defined as the time from the start of regorafenib to death. Progression-free survival (PFS) was defined as the time from the start of regorafenib therapy to disease progression. OS and PFS were evaluated using Kaplan–Meier method and compared using the log-rank test. Univariate and multivariate Cox proportional hazard regression models were used to analyze factors associated with survival. A *P* value of < 0.05 was considered statistically significant. All statistical analyses were performed using SPSS statistical software (version 26; SPSS-IBM, Chicago, IL, USA).

## Results

### Patient characteristics and regorafenib treatment

A total of 58 patients who received the regorafenib treatment were included in this study. The baseline characteristics of patients are presented in Table 1. The median age of the patients was 60 years, and most patients (*n* = 53,91.4%) were men. The Child-Pugh scores were 5 and 6 in 65.5 and 34.5% of patients, respectively. A total of 15 patients were Barcelona Clinic Liver Cancer (BCLC) stage B and 43 patients as BCLC C. Vascular invasion was noted in 18 (31.0%) patients and extrahepatic metastasis in 36 (62.1%) patients.

**Table 1:** Baseline characteristics of the patients

	***N*** = 58
Age (years)	60 (33–86)
Sex, male	53 (91.4)
Etiology of HCC	
HBV	
Others	
Child-Pugh score	
A5	38 (65.5)
A6	20 (34.5)
ALBI grade	
1	26 (44.8)
2	32 (55.2)
AFP	594 (10–9242)
PIVKA-II	4899 (796–25,522)
BCLC stage	
B	15 (25.9)
C	43 (74.1)
Vascular invasion (yes)	18 (31.0)
Extrahepatic meta (yes)	36 (62.1)
Lung	20 (34.5)
Lymph node	10 (17.2)
Bone	8 (13.8)
Adrenal gland	2 (3.4)
Others	9 (15.5)
Sorafenib treatment duration (months)	2.9 (1.5–28.1)

Values are presented as median (interquartile range) or number (%) of patients

*ALBI* albumin–bilirubin, *HCC* hepatocellular carcinoma, *HBV* hepatitis B virus, *BCLC* Barcelona Clinic Liver Cancer, *AFP* alpha-fetoprotein, *PIVKA* protein induced by vitamin K absence or antagonist

The median duration of regorafenib administration was 2 months (range: 0.5–19 months). All patients experienced at least one treatment-related adverse event. However, no treatment-related deaths was noted. Among the 28 patients who showed progression on regorafenib, 18 (64.3%) were treated with nivolumab, one with transarterial chemoembolization (TACE), and two with radiotherapy. We evaluated the best tumor response in 51 patients with at least one follow-up radiologic assessment. Six (11.8%) patients achieved partial response (PR) and 25 (49.0%) patients achieved stable disease (SD). PD was the best response in 20 (39.2%) patients. The disease control rate (DCR) was 60.8%.

### Survival analysis and predictors of PFS and OS

The median follow up duration was 5 months. The median OS was 8 months (95% confidence interval [CI] = 5.761–10.239), and the median PFS was 3 months (95% CI = 0.981–5.019). The median times from the start of sorafenib to death was 13 months (95% CI = 5.4–20.6). Table 2 summarizes the results of factors associated with OS. The AFP level (hazard ratio [HR] = 2.750, 95% CI = 1.259–6.010, *P* = 0.011) and SII (HR = 2.211, 95% CI = 1.089–4.489, *P* = 0.028) were found to be independent predictors of poor OS in multivariate analysis. Although we also analyzed independent predictors of PFS, no predictive factors were found to be associated with PFS (data not shown).

**Table 2:** Univariate and Multivariate COX Regression for Overall Survival

Variables	Univariate		Multivariate	
	*P* value	HR(95% CI)	*P* value	HR(95% CI)
Age	0.277	0.684(0.345–1.357)		
NLR ≥ 2.4	0.362	1.375(0.694–2.725)		
PLR ≥ 108	0.098	1.783(0.898–3.539)		
SII ≥ 340	0.020	2.308(1.140–4.676)	0.028	2.211(1.089–4.489)
Child Pugh score 6	0.248	1.549(0.737–3.254)		
ALBI grade 2	0.055	2.004(0.987–4.072)		
AFP ≥ 400 ng/mL	0.008	2.849(1.309–6.200)	0.011	2.750(1.259–6.010)
PIVKA-II ≥ 1000 mAU/mL	0.079	2.122(0.917–4.913)		
BCLC stage C	0.527	0.787(0.375–1.652)		
Vascular invasion	0.171	1.652(0.805–3.391)		
Extrahepatic metastasis	0.450	0.770(0.390–1.518)		

*AFP* alpha-fetoprotein, *ALBI* albumin–bilirubin, *BCLC* Barcelona Clinic Liver Cancer, *NLR* neutrophil-to-lymphocyte ratio, *PLR* platelet-to-lymphocyte ratio, *PIVKA* protein induced by vitamin K absence or antagonist, *SII* systemic immune-inflammation index

### Prediction of survival using SII

**Fig. 1:**
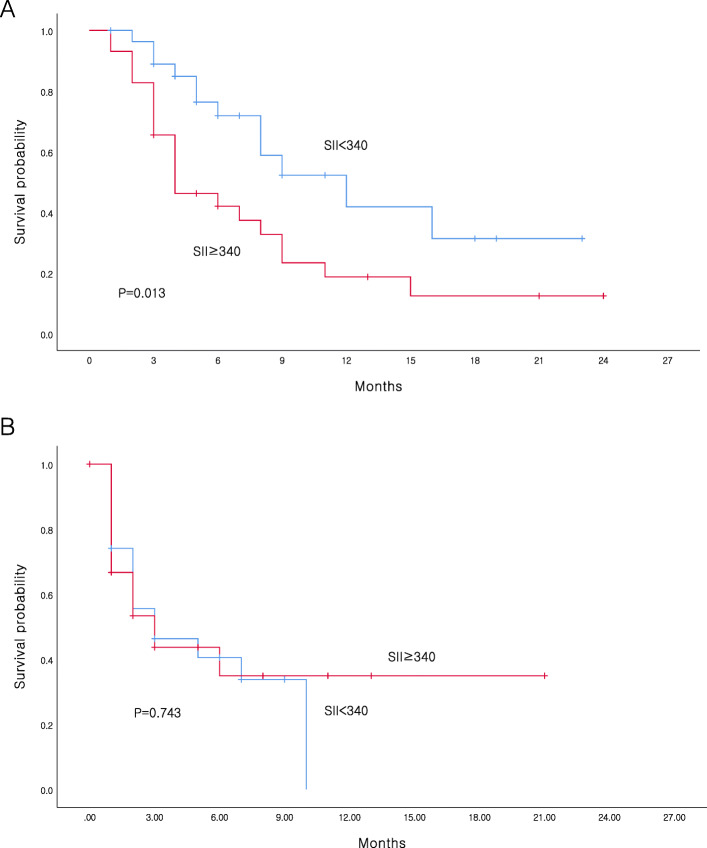
**a** Overall survival and **b** Progression free survival based on systemic immune-inflammation index

**Fig. 2:**
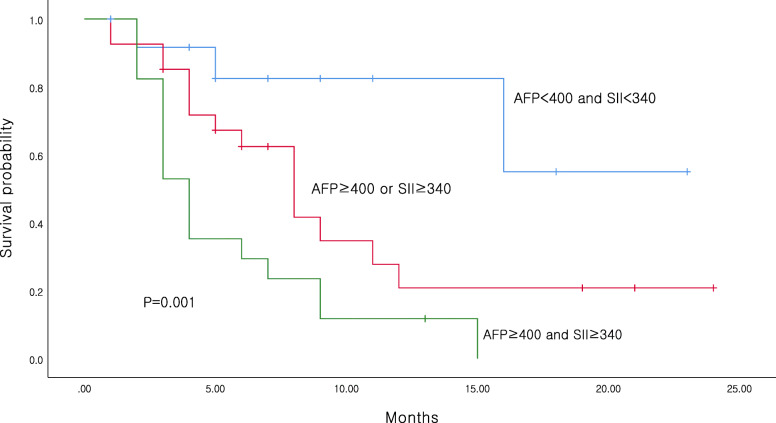
Overall survival based on different levels of systemic immune-inflammation index and alpha-Fetoprotein

The OS was significantly lower in patients with high SII (≥340) than in patients with low SII (*P* = 0.013; Fig. 1a). No difference was noted in PFS between patients with low and high SII (*P* = 0.743; Fig. 1b). We analyzed patient OS using factors that were significant in multivariate analysis, which were SII and AFP level. Categorization of patients based on the SII and AFP level revealed that patients with high SII and AFP level had significantly lower OS than those with low SII and AFP level (*P* = 0.001; Fig. 2). The correlation values between the SII and clinical features are presented in Table 3. We found that high SII levels were associated with more advanced BCLC stage (*P* = 0.036; Table 3).

**Table 3:** Clinical factors associated with SII

Variables	Low SII (***n*** = 29)	High SII (***n*** = 29)	***P*** value
Age			0.599
< 60	15 (51.7)	13(44.8)	
≥ 60	14(48.3)	16(55.2)	
Sex			0.640
Male	26(89.7)	27(93.1)	
Female	3(10.3)	2(6.9)	
Child Pugh score			0.401
A5	18(62.1)	21(72.4)	
A6	11(37.9)	8(27.6)	
ALBI grade			1.000
1	13(44.8)	13(44.8)	
2	16(55.2)	16(55.2)	
AFP (ng/mL)			0.597
< 400	14(48.3)	12(41.4)	
≥ 400	15(51.7)	17(58.6)	
PIVKA-II (mAU/mL)			0.240
< 1000	10(34.5)	6(20.7)	
≥ 1000	19(65.5)	23(79.3)	
NLR			0.000
< 2.4	23(79.3)	5(17.2)	
≥ 2.4	6(20.7)	24(82.8)	
PLR			0.000
< 108	12(79.3)	6(20.7)	
≥ 108	6(20.7)	23(79.3)	
BCLC stage			0.036
B	11(37.9)	4(13.8)	
C	18(62.1)	25(86.2)	
Vascular invasion			1.000
No	20(69.0)	20(69.0)	
Yes	9(31.0)	9(31.0)	
Extrahepatic metastases			0.104
No	14(48.3)	8(37.6)	
Yes	15(51.7)	21(72.4)	

Values are presented as number (%) of patients

*AFP* alpha-fetoprotein, *ALBI* albumin–bilirubin, *BCLC* Barcelona Clinic Liver Cancer, *NLR* neutrophil-to-lymphocyte ratio, *PLR* platelet-to-lymphocyte ratio, *PIVKA* protein induced by vitamin K absence or antagonist, *SII* systemic immune-inflammation index

## Discussion

Recent promising results of new systemic treatments, including tyrosine kinase inhibitors and immunotherapy, have enabled patients with advanced HCC to receive multiple systemic treatments in sequence. Regorafenib, an oral multikinase inhibitor, demonstrated survival benefit over placebo in the phase 3, double-blinded RESORCE study. An exploratory analysis in the RESORCE trial revealed that sorafenib-regorafenib sequential treatment could provide extended survival (> 2 years) to patients with advanced HCC who did not benefit from locoregional theray [19]. After adopting of regorafenib as an initial second-line agent, several real-life reports with good results of regorafenib treatment when progressed on sorafenib were published. Recently, several novel therapeutic agents as second-line therapies after sorafenib failure have shown promising results. Several treatment options are currently available for patients who failed on sorafenib treatment. Therefore, it is important to identify factors to predict treatment response or prognosis.

The present study investigated the predictive factors using systemic inflammatory markers by evaluating the efficacy and safety of sorafenib-regorafenib sequential therapy in patients with advanced HCC. This study revealed that regorafenib was well tolerated and favorable safety in patients with advanced HCC. Regorafenib demonstrated an objective response rate (ORR) of 11.8%, DCR of 60.8%, a median PFS of 3 months, and a median OS of 8 months. These results are similar to those of previous studies [20]. We investigated the prognostic role of systemic inflammatory markers including SII and found that the SII was an independent predictive factor associated with OS. High SII levels at initiation of regorafenib were associated with poor survival.

Previous studies have demonstrated association of inflammation markers with cancer prognosis and elevated SII is associated with poor OS or PFS of patients with cancers. In HCC, many studies have reported relationship between SII and prognosis of patients treated with various treatments [21–23]. As a combined score based on peripheral platelet, lymphocyte and neutrophils counts, the predictive value of SII for survival may be explained by the role of these immune cells. High SII usually results from thrombocytosis, neutrophilia and lymphopenia, suggesting a decreased immune response. Inflammation plays essential role in the development of cancer and promotes all stage of tumor progression. Cancer cells are surrounded by stromal cells and immune cells to form tumor microenvironment [24]. Increasing evidences have demonstrated that neutrophilia and thrombocytosis are associated with cancer progression [25–28]. Neutrophils play pro-tumoral roles through multiple mechanisms. Neutrophils can enhance cancer cell invasion, proliferation and metastasis by releasing inflammatory mediators such as neutrophil elastase, matrix metalloproteinase-9, and interleukin-8. Neutrophils secrete the pro-inflammatory factors in the tumor microenvironment, resulting in lymphocyte apoptosis and immunosuppression [29]. Platelets act as multifunctional cells participating in hemostasis, tissue generation and immune response as well as in cancer growth, invasion, and metastasis. Growing evidence had demonstrated that platelets facilitate cancer progression and have a well-defined role in cancer invasion and metastasis [30, 31]. Conversely, lymphocytes are known to play a fundamental role in cell-mediated immune response against cancer. Lymphopenia, which reflects the decreased immune surveiilance against cancer, has been reportedly to be associated with poor survival in various solid tumors [32–34]. We hypothesized that cancer therapeutic agents cause immune perturbation and an inflammation-based prognostic factor can reflect a patient’s systemic immune status. Thus, due to high platelets and neutrophils levels while low lymphocytes level, a high SII reflected a weak immune response in patients that favor pro-tumoral microenviroment.

The AFP level has been used as a diagnostic criterion and is well known to be correlate with HCC prognosis. High AFP levels are associated with larger tumor, bilobar involvement, vascular invasion, poorly differentiated histology and decreased survival [35]. The AFP level has been included in several HCC prognostic scoring systems [36–38]. High AFP levels are recognised as a poor prognostic factor, and AFP level higher than 400 ng/mL has been consistently associated with poor prognosis in several HCC treatments [39, 40]. These results may be elucidated from the relationship between AFP level and VEFGR expression level. VEGF and VEGFR-2-mediated signaling play important roles in angiogenesis and contribute to tumor growth in various cancers, including HCC [41, 42]. Increased AFP levels have been associated with increased VEGFR expression and increased angiogenesis in HCC [43, 44]. In this study, patients with high AFP and SII showed poor prognosis compared with those with low AFP level and SII. However, no correlation was found between the AFP and SII.

Despite the survival benefit, the overall response of regorafenib in patients with HCC who progressed on sorafenib treatment are modest and heterogenous because the prognosis of HCC is affected by tumor stage, severity of underlying liver disease and performance status. Moreover, the heterogeneity of the tumor staging itself can lead to a various prognosis. Although tumor staging is an important predictor for prognosis, a more complete understanding of tumor biology and host immune status are required to predict treatment response. This study investigated factors including well-known variable in previous studies, such as tumor staging and tumor markers associated with survival. Our study confirmed that there is still a relevant discrepancy between tumor staging and prognosis, raising the importance of understanding tumor biology and host immune status.

This study has several limitations. First, this study was a retrospective, single-center study with a small number of patients. Second, cutoff value of SII is arbitrary and validation of the cutoff value was not performed. Third, we did not conduct additional experiments to identify the underlying mechanism of the relationship between the SII and survival in patients with advanced HCC received regorafenib treatment. However, despite these limitations, this is the first study according to our knowledge to suggest that SII alone can predict the prognosis of patients with advanced HCC who received sorafenib-regorafenib sequential treatment.

## Conclusion

Our data demonstrated that elevated SII is associated with poor OS as well as the possibility of using the AFP level combined with the SII in patients with HCC who received sorafenib-regorafenib sequential treatment. If validated in a larger prospective study, the SII may provide a simple method for identifying patients with poor prognosis. Early identification of this poor prognosis group can provide an opportunity to change treatment strategies to improve patient outcomes.

## Data Availability

The datasets used and/or analysed during this study are available from the corresponding author on reasonable request.

## Abbreviations

AFP: Alpha-Fetoprotein
BCLC: Barcelona Clinic Liver Cancer
HCC: Hepatocellular carcinoma
NLR: Neutrophil to lymphocyte ratio
PFS: Progression free survival
PLR: Platelet to lymphocyte ratio
OS: Overall survival
PIVKA: Protein induced by vitamin K absence or antagonist-II
SII: Systemic immune-inflammation index
